# L’ostéotomie de Chiari dans la prise en charge de la dysplasie de la hanche chez l’adulte: à propos de 9 cas

**DOI:** 10.11604/pamj.2013.14.65.2101

**Published:** 2013-02-16

**Authors:** Mohammed Shimi, Hicham Mahdane, Atif Mechchat, Abedelhalim El Ibrahimi, Abedelmajid El Mrini

**Affiliations:** 1 Service de chirurgie ostéoarticulaire B4 CHU Hassan II Fès, Maroc

**Keywords:** Hanche douloureuse, dysplasie, Chiari, painful hip, dysplasia, Chiari

## Abstract

La dysplasie acétabulaire de l’adulte jeune entraîne, dans plus de 50% des cas, une coxarthrose secondaire avant l’âge de 50 ans, l’ostéotomie de CHIARI a été décrite initialement dans le traitement de la dysplasie acétabulaire de l’enfant et de l’adolescent, elle a vu ses indications s’étendre à la dysplasie acétabulaire de l’adulte. Nous avons réalisé 9 ostéotomies de CHIARI de 2009 à 2012. Les 9 hanches ont été évaluées cliniquement et radiologiquement en préopératoire et en postopératoire, avec un recul moyen de 18.4 mois. L’ostéotomie a été réalisée sur des hanches douloureuses dysplasiques, sans arthrose (45%) ou avec une arthrose peu évoluée (stade 2: 11%) ou évoluée (stade 3 et 4: 44%). Les résultats fonctionnels ont été très satisfaisants au dernier recul. En effet, le score PMA au dernier recul était de 17.4 en moyenne, avec en particulier, une action antalgique remarquable. Radiologiquement, l’ostéotomie a normalisé pratiquement dans tous les cas la coxométrie, grâce à une médialisation importante habituellement supérieure à 20 mm (87.5%). L’ostéotomie de CHIARI est une intervention sûre. Si l’indication est correctement posée, elle soulage remarquablement les patients et stoppe l’arthrose. Elle garde donc une place privilégiée dans le traitement de la coxarthrose même évoluée sur dysplasie acétabulaire pure ou mixte.

## Introduction

La dysplasie acétabulaire de l’adulte jeune entraîne, dans plus de 50% des cas, une coxarthrose secondaire avant l’âge de 50 ans [1]. Malgré les progrès de l’arthroplastie, la prothèse totale de la hanche du sujet jeune ne semble pas être pleinement satisfaisante en termes de durée de vie [2]. Elle est également de réalisation difficile sur une hanche dysplasique. La chirurgie conservatrice est alors une alternative de choix chez l’adulte jeune. Parmi ces interventions conservatrices, l’ostéotomie de Chiari. Le but de notre travail est d’évaluer les résultats obtenus par ce type d’intervention dans le traitement des dysplasies acétabulaires de l’adulte et d’étudier son intérêt dans l’amélioration fonctionnelle.

## Méthodes

### La série

De 2009 à 2012, 9 ostéotomies de CHIARI ont été réalisées chez 7 patients. Il s’agissait de 5 femmes et de 2 hommes, l’âge moyen lors de l’intervention était de 28,8 ans (extrêmes: 17 - 48 ans), l’indice de masse corporelle (IMC) moyen était de 28.6 kg/m^−2^ (extrêmes: 22 - 35 kg/m^−2^), 3 patients étaient obèses, le recul moyen de cette série était de 18.4 mois.

Les antécédents de ces 9 hanches étaient:

- 2 dysplasies séquellaires de luxation congénitale de la hanche réduites orthopédiquement dans l’enfance.
- 1 luxation traumatique de la hanche chez un patient à l’âge de 18ans, traitée orthopédiquement.
- 6 dysplasies de la hanche découvertes à l’âge adulte.
- La hanche controlatérale était normale dans 5 cas.

### L’évaluation préopératoire

**Figure 1:**
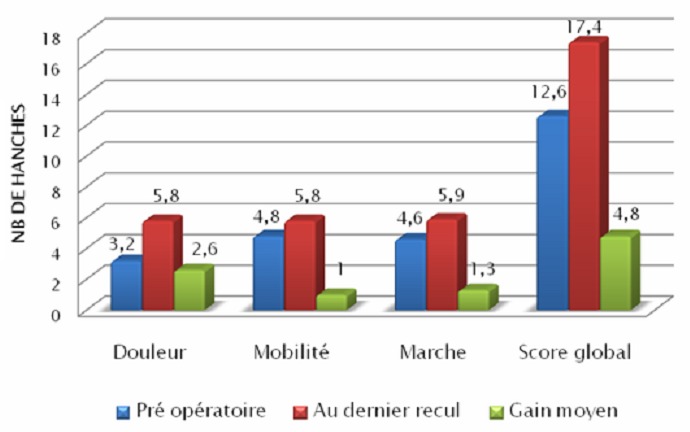
Évaluation clinique par le PMA en préopératoire et au dernier recul

Sur le plan clinique: L’état fonctionnel des 9 hanches a été quantifié par le score de Postel Merle D’Aubigner (score PMA) [3]: La gêne fonctionnelle était sévère, puisque 4 hanches étaient cotées médiocre (44%) et 5 hanches étaient cotées passables (56%). La cotation globale préopératoire était en moyenne de 12,6 (Figure 1).

**Figure 2:**
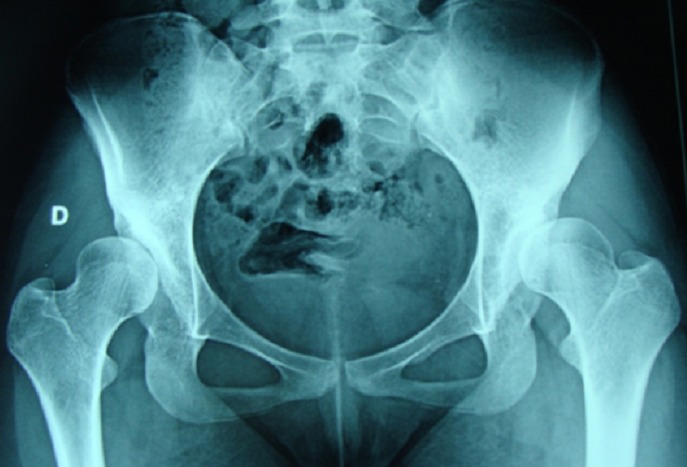
Dysplasie acétabulaire extrême bilatérale, chez une patiente de 17 ans (hanche droite: VCE -6°, hanche gauche: VCE –10°)

L’évaluation Radiologique a été réalisée sur des radiographies standards comprenant deux incidences en charge: bassin de face et le faux profil de Lequesne [4]. L’analyse radiologique par coxométrie a permis d’apprécier la sévérité de la dysplasie acétabulaire et/ou fémorale, l’état de la tête fémorale et l’existence d’une coxarthrose (Figure 2). Les hanches ont été classées ainsi selon la classification du Hip Study Group [5], Dans notre série: 1 hanche (11.1%) était classée comme dysplasie moyenne, 6 hanches (66.7%) comme dysplasie sévère, et 2 hanches (22.2%) comme dysplasie extrême (Tableau 1). La tête fémorale était régulière chez 4 patients (44.4%), irrégulière chez 3 patients (33.3%), et aplatie chez 2 patients (22.3%). La valeur moyenne de l’angle cervico-diaphysaire CC’D dans notre série était de 141,9° (extrêmes: 128°; 165°). La répartition préopératoire du stade d’arthrose selon Mourgues et Pattes était: stade 0 dans (45%), une arthrose peu évoluée (stade 2: 11%) ou évoluée (stade 3 et 4: 44%).

**Tableau 1:** Valeurs angulaires moyennes en pré, postopératoire immédiat et au dernier recul, avec le gain moyen

	VCE	VCA	HTE	Indice de WAGNER
Préopératoire	9.3° [-10°; 21°]	22° [1 cas]	24,7° [11° ; 49°]	53.6% [29% ; 70%]
Postopératoire immédiat	29.7° [15° ; 44°]	-	15.2° [11° ; 23°]	84% [70% ; 98%]
Dernier recul	32.9° [22° ; 46°]	32.7° [3 cas]	13.1° [10° ; 20°]	90% [80% ; 100%]
Gain moyen	24.3° [17° ; 36°]	-	11.6° [0 ; 35°]	36.4% [25% ; 53%]

VCE : Angle de couverture externe ; VCA : Angle de couverture antérieure ; HTE : Angle d’obliquité du toit

**Figure 3:**
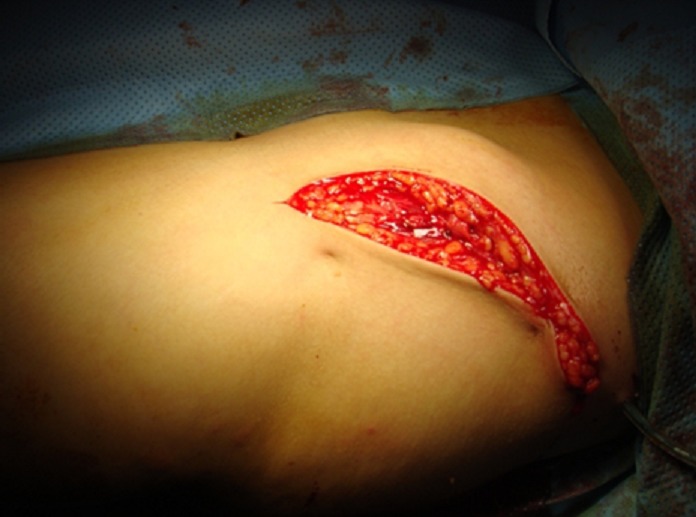
Voie d’abord comportant uniquement la partie exopelvienne de la voie de Smith Peterson

La technique opératoire:

- Tous les patients ont été opérés sous anesthésie rachidienne, en décubitus dorsal sur table orthopédique et contrôle par amplificateur de brillance, un appui pubien doit est placé à distance de la face médiale de la cuisse à opérer de façon à ne pas gêner la médialisation ultérieure.
- Nous avons utilisé une voie d’abord mini invasive, comportant uniquement la partie iliaque externe de la voie de SMITH-PETERSEN. L’abord était essentiellement exopelvien (Figure 3). La région supra-acétabulaire est exposée jusqu’à la grande échancrure sciatique après désinsertion de l’éventail des muscles fessiers.
- Le trait de l’ostéotomie est guidé par la mise en place d’une broche de 20/10 sous contrôle de l’amplificateur de brillance. L’ostéotomie est effectuée d’avant en arrière, au moyen de ciseaux frappés.
- La médialisation se produit dès le membre mis en abduction, La fixation de l’ostéotomie est faite par vis. Un drainage est laissé en place durant en moyenne 2 jours.

## Résultats

**Figure 4:**
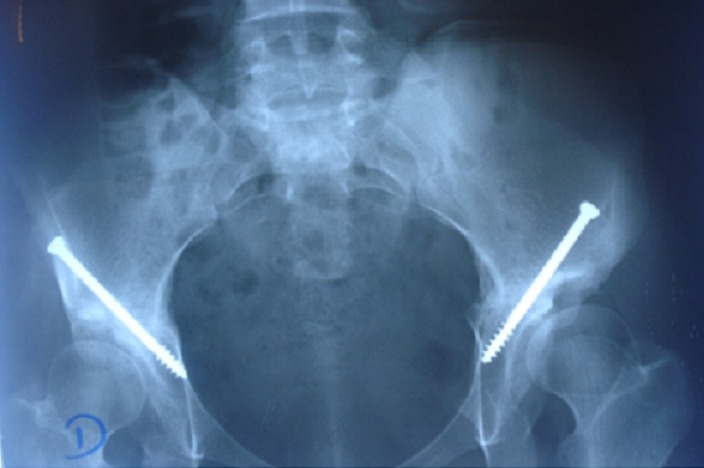
Score Postel Merle d’Aubigné (PMA): 18. Coxométrie: angles de couverture externe (VCE): 23°, angles de couverture antérieur (VCA) 33°, angle d’obliquité du toit (HTE) 12°. Indice de WAGNER 85%

L’analyse des résultats en postopératoire immédiat et au dernier recul a été réalisée par le score PMA et sur des radiographies du bassin de face. En dernier recul, les valeurs de la coxométrie étaient proches de la normale (Figure 4), l’amélioration la plus sensible se faisant au niveau de l’angle de couverture externe VCE et l’angle de couverture antérieure VCA (Tableau 1). Le résultat fonctionnel global selon la cotation PMA, au recul moyen de 18.4 mois, était excellent dans 55,5% des cas (5 cas), très bon dans 33.3% des cas (3 cas) et bon dans 11.2% des cas (1 cas) (Figure 1).

Pour l’ensemble de la série, nous n’avons recensé que deux complications concernant 2 patients (22.2%): Une complication per-opératoire: une vis intra-articulaire, la vis a été enlevé après consolidation et un patient avait une boiterie modérée sans douleur à un recul de 12 mois.

## Discussion

L’ostéotomie de Chiari a été décrite par Karl Chiari en 1955 dans le traitement de la dysplasie acétabulaire de l’enfant et de l’adolescent, et elle a vu ses indications s’étendre à la dysplasie acétabulaire de l’adulte [6, 7]. C’est une intervention qui reste d’actualité et qui donne de bons résultats fonctionnels avec un recul de plus de 20ans chez certains auteurs.

Notre étude a confirmé l’effet spectaculaire de l’ostéotomie de Chiari sur la douleur, Le score PMA au dernier recul est amélioré avec un gain moyen global de 4,8 points, Nakata et AL [8] confirment la persistance de l’amélioration fonctionnelle malgré le grand recul, en effet, ils trouvent un score PMA de 16,8 à 5 ans, 17 à 10 ans et 16,6 à 13 ans. Par contre, Hulet et AL [9] montrent une stabilité du résultat fonctionnel entre 5 et 10 ans, puis une dégradation après 10 ans. Les résultats semblent meilleurs chez les patients dont l’âge est inférieur à 30 ans [9], et La qualité technique de l’intervention est l’un des paramètres qui influencent le plus le résultat [10]. La bonne réalisation du contrat biomécanique de l’ostéotomie de Chiari est réputée difficile [11]. Les couvertures supérieures et antérieures sont améliorées de manière statistiquement significative en postopératoire et se maintiennent dans le temps [12].

Nous utilisons, la voie exopelvienne de Smith Peterson (mini invasive), nous nous dispensons de l’abord endopelvien: celui-ci ne nous semble pas indispensable pour contrôler l’ostéotomie. Les chefs du muscle psoas iliaque protègent les grands axes vasculaires plus médiaux et préservent la vascularisation osseuse. Cette intervention, bien maîtrisée techniquement, est source de peu de complications [13]. Nous n’avons pas eu à déplorer de nécrose du toit du cotyle ni de lésions du nerf sciatique [6, 14].

Certains auteurs considèrent que l’arthrose évoluée est un facteur pronostic péjoratif au point qu’ils considèrent qu’une ostéotomie de Chiari ne devrait pas être réalisée chez des patients avec une arthrose avancée [7, 9, 10], nous pensons comme Duquennoy [15], qu’une arthrose évoluée n’est pas une contre indication à l’ostéotomie de Chiari, si elle est la conséquence d’une dysplasie acétabulaire sévère, Yasunaga et AL [16] publient avec un recul moyen de 8,5 ans, l’absence de progression de l’arthrose dans 72,2% des cas à 10 ans.

La reprise chirurgicale par prothèse ne pose pas de problème particulier. Au contraire, l’amélioration de la couverture antérieure et supérieure simplifie l’intervention. Kempf et Persoons [17] retrouvent sur 23 prothèses un seul cas ayant nécessité une greffe osseuse de recentrage. Hashemi-Nejad et AL [18] ont comparé 28 arthroplasties sur ostéotomie de Chiari à un groupe témoin de 50 arthroplasties réalisées chez des patients ayant une dysplasie de hanche. Ils retrouvent un temps opératoire plus court et moins de complications précoces dans le groupe ostéotomie de Chiari avec un résultat fonctionnel équivalent à moyen terme. Ils en concluent que l’ostéotomie de Chiari permet de repousser la date de l’arthroplastie en facilitant la pose de la future prothèse sans compromettre le résultat fonctionnel.

## Conclusion

Quoique délicate à bien réaliser, l’ostéotomie de Chiari est une intervention sûre. Si l’indication est correctement posée, elle soulage remarquablement les patients et stoppe l’arthrose. Dans tous les cas, elle permet de reculer l’échéance de la prothèse totale de la hanche, et de préparer idéalement bien à l’implantation du composant cotyloïdien de la prothèse, sans compromettre la mise en place du composant fémoral. Elle garde donc une place privilégiée dans le traitement de la coxarthrose même évoluée sur dysplasie acétabulaire pure ou mixte.
